# P277: Situation analysis of patient safety and infection control in health institutions in postconflict in DR Congo

**DOI:** 10.1186/2047-2994-2-S1-P277

**Published:** 2013-06-20

**Authors:** EN Dunia, AM Kady

**Affiliations:** 1 RIPAQS, Kisangani,The Democratic Republic of the Congo; 2 RIPAQS, Kinshasa, The Democratic Republic of the Congo

## Introduction

The State of infections acquired during care procedures whose prevalence rate is estimated at about 15% in some benchmark institutions reflects the alarming situation of provision of care and patient safety in DR Congo.

## Objectives

Conduct an inventory of patient safety in health facilities in the city Kinshasa.

## Methods

Study conducted using survey and observation checklist covering the different aspects of patient safety: institutional, medical technical equipment, medical devices, blood products, drugs, biological graft, waste management medical needles and treatment materials.

## Results

At the institutional level: the lack of standards and policies on patient safety except on injection safety; lack of policy on the fight against nosocomial infections; reporting system for adverse events.

Technically: Absence of risk management procedures, training non-existent on the quality and safety of patients in Kinshasa (90% of staff) and 89% of patients do not know their rights and lack of association of patient safety care.

## Conclusion

The implementation of a policy of promotion of hospital hygiene and the fight against adverse events associated with their care requires a partnership challenging multiple states, civil society, communicators, the private sector and international institutions.

## Competing interests

None declared.

